# Tissue-specific patterns of regulatory changes underlying gene expression differences among *Ficedula* flycatchers and their naturally occurring F_1_ hybrids

**DOI:** 10.1101/gr.254508.119

**Published:** 2020-12

**Authors:** Carina F. Mugal, Mi Wang, Niclas Backström, David Wheatcroft, Murielle Ålund, Marie Sémon, S. Eryn McFarlane, Ludovic Dutoit, Anna Qvarnström, Hans Ellegren

**Affiliations:** 1 Department of Ecology and Genetics, Uppsala University, 752 36 Uppsala, Sweden;; 2 Department of Zoology, Stockholm University, 106 91 Stockholm, Sweden;; 3 Department of Integrative Biology, Michigan State University, East Lansing, Michigan 48824, USA;; 4 ENS de Lyon, Laboratory of Biology and Modelling of the Cell, Lyon University, 69364 Lyon Cedex 07, France;; 5 Institute of Evolutionary Biology, University of Edinburgh, Edinburgh EH9 3FL, United Kingdom;; 6 Department of Zoology, University of Otago, Dunedin 9016, New Zealand

## Abstract

Changes in interacting *cis*- and *trans*-regulatory elements are important candidates for Dobzhansky-Muller hybrid incompatibilities and may contribute to hybrid dysfunction by giving rise to misexpression in hybrids. To gain insight into the molecular mechanisms and determinants of gene expression evolution in natural populations, we analyzed the transcriptome from multiple tissues of two recently diverged *Ficedula* flycatcher species and their naturally occurring F_1_ hybrids. Differential gene expression analysis revealed that the extent of differentiation between species and the set of differentially expressed genes varied across tissues. Common to all tissues, a higher proportion of Z-linked genes than autosomal genes showed differential expression, providing evidence for a fast-Z effect. We further found clear signatures of hybrid misexpression in brain, heart, kidney, and liver. However, while testis showed the highest divergence of gene expression among tissues, it showed no clear signature of misexpression in F_1_ hybrids, even though these hybrids were found to be sterile. It is therefore unlikely that incompatibilities between *cis*-*trans* regulatory changes explain the observed sterility. Instead, we found evidence that *cis*-regulatory changes play a significant role in the evolution of gene expression in testis, which illustrates the tissue-specific nature of *cis*-regulatory evolution bypassing constraints associated with pleiotropic effects of genes.

Following the seminal work of King and Wilson (1975) postulating that evolution occurs at two levels, the relative importance of changes in protein sequences and changes in gene expression has been a long-standing debate (Hoekstra and Coyne 2007; Wray 2007; Wagner and Lynch 2008). These two levels translate into the respective role of protein-coding sequences and gene regulatory sequences in adaptation and speciation. Methodological limitations have led to a bias in favor of studies of the former category since it is easier to identify and functionally annotate protein-coding sequences than regulatory sequences/elements. To circumvent these limitations inherent to the study of regulatory evolution, the role of regulatory changes has mostly been studied via analysis of gene expression (Khaitovich et al. 2005; Brawand et al. 2011; Sudmant et al. 2015). However, this is only an indirect approach since gene expression itself is a phenotype, which is not only genetically determined but also varies due to environmental conditions. To control for environmental variation, studies on gene expression evolution have therefore commonly been performed under laboratory conditions or common garden settings (Romero et al. 2012; Signor and Nuzhdin 2018).

Compared to regulatory changes, changes in protein-coding sequences are suggested to have greater pleiotropic effects and therefore to evolve under stronger selective constraint (Prud'homme et al. 2007; Wray 2007; Fraser 2011; Romero et al. 2012; Wittkopp and Kalay 2012). Likewise, changes in *trans*-regulatory elements are suggested to have greater pleiotropic effects than changes in *cis*-regulatory elements because individual *trans*-acting factors often bind to multiple *cis*-regulatory elements from multiple genes (Prud'homme et al. 2007). Changes in a single *cis*-regulatory element may be expected to have mostly targeted effects and could therefore play an important role in adaptation by bypassing the constraints associated with pleiotropic genes or *trans*-factors (Carroll 2005; Wray 2007). This has recently been exemplified for single traits, such as pigmentation in *Drosophila* (Hollocher et al. 2000; Kopp et al. 2000; Kopp and True 2002; Gompel and Carroll 2003; Gompel et al. 2005) and pelvic reduction in sticklebacks (Cresko et al. 2004; Shapiro et al. 2004; Morris et al. 2014; Marques et al. 2016).

On the other hand, accumulation of changes in interacting *cis*- and *trans*-regulatory elements may show reciprocal effects on gene expression level and thereby be compensatory. As a result, conservation of gene expression can be maintained at the same time as mutations in regulatory elements accumulate (Johnson and Porter 2000; Wittkopp and Kalay 2012). Because of this, regulation of gene expression may be of particular relevance to speciation where the interaction between divergent *cis*- and *trans*-regulatory elements is a candidate process for the formation of Dobzhansky-Muller hybrid incompatibilities (Haerty and Singh 2006; Hochholdinger and Hoecker 2007; Mack and Nachman 2017; Signor and Nuzhdin 2018). Specifically, previous studies suggest that incompatible interactions between *cis*- and *trans*-factors from hybridizing species would lead to misexpression of genes and hybrid dysfunction (Signor and Nuzhdin 2018) and contribute to intrinsic postzygotic reproductive isolation (Landry et al. 2005; Haerty and Singh 2006; Tulchinsky et al. 2014; Mack et al. 2016).

A powerful approach to study regulatory evolution and its role in hybrid misexpression is the analysis of allele-specific expression (ASE) in F_1_ hybrids (Wittkopp et al. 2004). By identifying the parent-of-origin of hybrid alleles, it is possible to differentiate between *cis*- and *trans*-effects. Crosses of inbred strains/species with substantially divergent genomes benefit the analysis as they show small within- but large between-species divergence. So far, studies on regulatory evolution have thus primarily focused on model organisms in lab settings (Wittkopp et al. 2004, 2008; Tirosh et al. 2009; Emerson et al. 2010; McManus et al. 2010; Goncalves et al. 2012; Schaefke et al. 2013; Coolon et al. 2014; Mack et al. 2016; Glaser-Schmitt et al. 2018), and the question has not been thoroughly investigated in natural populations. Therefore, it remains unclear if findings observed in lab settings can directly be transferred to the wild, where polymorphism is frequent and gene expression differences have arisen under environmental influence.

Here, we analyze regulatory sequence evolution in *Ficedula* flycatchers, which are well-known species in the field of speciation research. Collared flycatchers (*F. albicollis*) and pied flycatchers (*F. hypoleuca*) diverged approximately 1 MYA but co-occur in natural hybrid zones such as the Swedish island of Öland (Saetre et al. 1999; Qvarnström et al. 2010). Their F_1_ hybrids suffer from low to totally compromised fertility (Alatalo et al. 1990; Ålund et al. 2013), low attractivity (Alatalo et al. 1982; Svedin et al. 2008), and abnormal metabolic rate (McFarlane et al. 2016). By retrieving transcriptomic data across multiple tissues from naturally occurring F_1_ hybrids and their parental species, we studied hybrid gene expression in a setting relevant to speciation. We further used a novel statistical approach for detection of ASE particularly developed for natural populations (Wang et al. 2018) and also studied signatures of differentiation in candidate *cis*-regulatory elements. With the help of these complementary approaches, we investigated the role of regulatory changes underlying the evolution of gene expression and Dobzhansky-Muller hybrid incompatibilities between divergent regulatory elements.

## Results

We generated RNA-seq data from five collared flycatcher males and five pied flycatcher males, from five different tissues, with an average of 49 and 42 million raw reads per individual and tissue, respectively (Supplemental Table S1). In addition, data was also obtained from three male putative F_1_ hybrids with an average of 43 million raw reads per individual and tissue (Supplemental Table S1). Species and hybrid identity were confirmed genetically based on fixed differences between collared flycatcher and pied flycatcher (see Methods). Phylogenetic analysis of mtDNA sequences revealed that all three F_1_ hybrids were the result of crosses between a female pied flycatcher and a male collared flycatcher (Supplemental Fig. S1).

### Tissue-specific patterns of gene expression evolution

**Figure 1.**
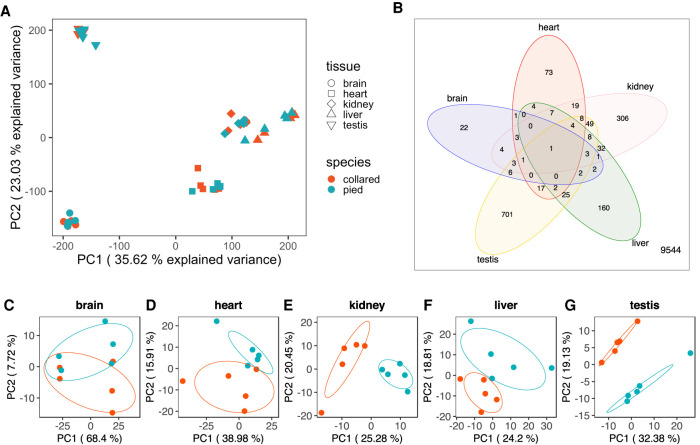
Variation in gene expression across tissues, and within and between species. (*A*) Principal component analysis (PCA) plot of the first two principal components of variation in gene expression across five different tissues for five collared flycatchers (red) and five pied flycatchers (blue). (*B*) Venn diagram of differentially expressed genes across the five tissues. Numbers indicate the number of genes in each category. The number of genes not differentially expressed in any of the tissues is provided in the *bottom right* corner. (*C*–*G*) PCA plots of the first two principal components of variation in gene expression separately for the five different tissues. Ellipses indicate the species-specific mean with a range of ±1 standard deviation, in red for collared flycatchers, in blue for pied flycatchers.

Principal component analysis (PCA) of gene expression patterns across tissues and species showed that the main source of gene expression variability was attributed to differences among tissues rather than species identity (Fig. 1A). This finding was confirmed quantitatively by between-groups PCA. While the variance associated with tissue identity was 86%, only a nonsignificant proportion of the variance (<1%) was associated with species identity (Monte-Carlo *P*-value = 0.88 for a sample size of 99). The latter observation is not unexpected in light of the recent divergence of collared flycatcher and pied flycatcher. However, after controlling for tissue identity, we found a significant association with species identity of 4.66% (Monte-Carlo *P*-value = 0.01 for a sample size of 99).

PCA for each tissue separately revealed variation among individuals from the same species (Fig. 1C–G). This could either reflect true biological variation or technical variation, such as differences in RNA quality across tissues. Nevertheless, variation between individuals from the same species was generally smaller than variation between individuals from each of the two species. The extent of differentiation between species varied across tissues (Fig. 1C–G). In line with the visual representation of the PCA (Fig. 1C–G), between-groups PCA revealed that species identity explained the highest amount of gene expression variation in testis (23.46%), followed by kidney (23.29%), liver (18.44%), heart (16.81), and brain (6.34%). In order to control for differences in RNA quality across tissues, we performed regression analysis of the amount of gene expression variation explained by species identity against the average RNA integrity number (RIN). Residuals were highest for testis (0.039), followed by kidney (0.034), heart (0.027), liver (0.017), and brain (−0.116). Thus, our main conclusion that testis shows the highest and brain the lowest expression divergence appears not to be caused by variation in RNA quality across tissues. This suggests that the rate of gene expression evolution indeed differs across tissues, consistent with observations in other species (Khaitovich et al. 2005; Brawand et al. 2011; Sudmant et al. 2015). Differential gene expression analysis confirmed this difference, with brain showing only 55 and testis as many as 1031 significantly differentially expressed (DE) genes (significance threshold FDR < 0.05). Intermediate numbers were detected for heart (160), liver (311), and kidney (537). The power of the differential gene expression analyses was of comparable strength across tissues (Supplemental Fig. S2 in Supplemental Analysis) and also of comparable strength to a recent RNA-seq study across four tissues in primates (Supplemental Fig. S3 in Supplemental Analysis).

In addition to different rates of expression evolution across tissues, the set of genes that were differentially expressed also differed among tissues (Fig. 1B). Only one gene, *DPP7*, which is likely to be nonfunctional in pied flycatchers due to a deletion (Uebbing et al. 2016), was differentially expressed across all five tissues. In contrast, at least 40% of the DE genes in each tissue were differentially expressed only in that tissue, here referred to as tissue-specific differential gene expression. Since a large proportion (12,467 out of 16,228) of the analyzed genes were expressed in all five tissues, and only few genes in just one tissue, the proportion of tissue-specific differential gene expression was larger than would be expected by chance. This suggests that broadly expressed genes are regulated by tissue-specific regulatory elements.

Common to all tissues, a higher proportion of Z-linked genes than autosomal genes showed differential expression (Table 1). Across tissues, the mean proportion of DE genes was 5.70% on the Z Chromosome compared to 3.19% for autosomes. The difference was statistically significant across all tissues, which provides evidence for a broad fast-Z effect in gene expression evolution.

**Table 1.** Numbers of differentially expressed (DE) genes and nondifferentially (nDE) expressed genes between collared flycatcher and pied flycatcher in five different tissues, separately for autosomes and the Z Chromosome

	Autosomes		Z Chromosome		
Tissue	DE	nDE	DE	nDE	*P*-value
Brain	41	12,427	11	531	**1.0 × 10^−7^**
Heart	127	10,832	14	463	**4.0 × 10^−4^**
Kidney	448	11,412	37	483	**7.3 × 10^−5^**
Liver	269	11,251	17	487	**4.8 × 10^−2^**
Testis	908	11,180	57	471	**2.1 × 10^−3^**

Only genes that could be assigned a chromosomal location and showed significant expression in the respective tissue are included in the list. Differences in the distribution of DE and nDE genes between autosomes and the Z Chromosome were assessed by a hypergeometric test. Significant differences (*P*-value < 0.05) are highlighted in bold and underlined if significance was retained after Holm-Bonferroni correction.

### Determinants of gene expression evolution

We performed generalized linear regression (GLM) analysis of differential gene expression (*n* = 9658 autosomal genes) using the number of protein–protein interactions (PPI), tissue specificity (τ), intraspecific variation in gene expression (ϕ), the ratios of nonsynonymous to synonymous diversity (π_N_/π_S_) and divergence (*d*_N_/*d*_S_), and genomic differentiation between the two flycatcher species (*F*_ST_) as candidate explanatory variables. The analysis was performed for each tissue separately, which revealed similarities as well as some interesting differences in key factors underlying gene expression evolution across tissues (Table 2). While τ was the major determinant of gene expression evolution in heart, kidney, liver, and testis, selective constraint on the protein sequence (*d*_N_/*d*_S_) was the major determinant in brain. The overall importance of τ for gene expression evolution clearly suggests that tissue-specific genes experience faster divergence of gene expression level, even though tissue-specific differential gene expression might also be achieved by tissue-specific regulation of broadly expressed genes (Fig. 1B). The role of selective constraint on the protein sequence in brain, on the other hand, supports the hypothesis that selective constraint on gene sequences and their level of expression might be coupled, at least in some tissues. *F*_ST_ was positively associated with differential gene expression in several tissues (kidney, liver, and testis), highlighting the importance of genomic background, and is possibly related to the degree of *cis*-regulatory variation.

**Table 2.** Generalized linear regression (GLM) analysis of differential gene expression between collared flycatcher and pied flycatcher against the number of protein–protein interactions (PPIs), tissue specificity (τ), intra-species variation in gene expression (ϕ), the ratio of nonsynonymous to synonymous diversity (π_N_/π_S_) and divergence (*d*_N_/*d*_S_), and genomic differentiation between collared flycatcher and pied flycatcher (*F*_ST_) as candidate explanatory variables separately for five different tissues

	Brain		Heart		Kidney		Liver		Testis	
	*t*-stat	*P*-value	*t*-stat	*P*-value	*t*-stat	*P*-value	*t*-stat	*P*-value	*t*-stat	*P*-value
PPI	0.33	7.38 × 10^−1^	−1.81	7.03 × 10^−2^	−0.50	9.60 × 10^−1^	−1.64	1.00 × 10^−1^	−0.52	6.04 × 10^−1^
τ	0.90	3.67 × 10^−1^	2.26	**2.37 × 10**^−**2**^	4.25	**2.11 × 10**^−**5**^	3.22	**1.26 × 10**^−**3**^	5.62	**1.88 × 10**^−**8**^
ϕ	−1.59	1.13 × 10^−1^	−1.87	6.21 × 10^−2^	−3.60	**3.23 × 10**^−**4**^	−3.19	**1.43 × 10**^−**3**^	−4.36	**1.33 × 10**^−**5**^
π_N_/π_S_	−0.12	9.03 × 10^−1^	0.84	3.98 × 10^−1^	−0.28	7.82 × 10^−1^	0.23	8.20 × 10^−1^	0.62	5.38 × 10^−1^
*d*_N_/*d*_S_	3.20	**1.39 × 10**^−**3**^	1.67	9.41 × 10^−2^	−0.52	6.04 × 10^−1^	2.36	**1.82 × 10^−2^**	1.66	9.66 × 10^−2^
*F*_ST_	0.34	7.35 × 10^−1^	1.05	2.95 × 10^−1^	3.48	**5.02 × 10**^−**4**^	3.15	**1.64 × 10**^−**3**^	4.79	**1.69 × 10**^−**6**^

For each tissue, the *t*-statistic of the association and *P*-values are reported. Significant associations (*P*-value < 0.05) are highlighted in bold and underlined if significance was retained after Holm-Bonferroni correction.

Multiple linear regression (MLR) analysis of log_2_-fold change against the same six candidate explanatory variables largely confirmed the GLM results (Supplemental Table S2), but MLR suggests stronger significance for determinants of gene expression evolution, while GLM appears to be more conservative.

### Gene expression in naturally occurring hybrids

**Figure 2.**
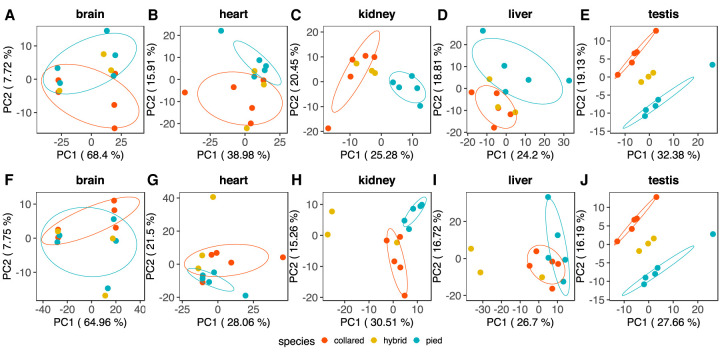
Principal component analysis plots of the first two principal components of variation in gene expression in five different tissues for five collared flycatchers (red), five pied flycatchers (blue), and three F_1_ hybrids (yellow). Ellipses indicate the species-specific mean with a range of ±1 standard deviation, in red for collared flycatchers, in blue for pied flycatchers. Note that a sample size of three does not allow computing ellipses for the F_1_ hybrids. (*A*–*E*) F_1_ hybrids are projected onto the PCA of the parental species. (*F*–*J*) F_1_ hybrids are directly included in the PCA.

To visualize the relative position of gene expression patterns of F_1_ hybrids compared to those of the parental species without letting potential misexpression in F_1_ hybrids blur the signal of evolutionary divergence between species, F_1_ hybrids were, in a first step, projected onto the PCA of collared flycatcher and pied flycatcher individuals (Fig. 2A–E). For brain, projection placed the F_1_ hybrids closer to pied flycatcher than to collared flycatcher, while the opposite trend was observed for heart, kidney, and liver. For testis, F_1_ hybrids were clearly placed between the parental species. These differences in clustering are suggestive of different modes of gene expression inheritance across tissues, that is, pied-dominant inheritance in brain, collared-dominant inheritance in heart, kidney, and liver, and additive inheritance in testis. Direct inclusion of F_1_ hybrids into the PCA revealed consistent inheritance patterns (Fig. 2F–J). However, separate clustering of F_1_ hybrids in heart, kidney, and liver further suggests that misexpression is abundant in these tissues. In testis, F_1_ hybrids still clearly clustered between the parental species, suggesting an absence of misexpression but additive inheritance in this tissue. Results for brain revealed little separation between F_1_ hybrids and their parental species, replicating the finding from the comparison of parental species that gene expression divergence is limited in brain.

Analyses of differential gene expression between hybrids and each parental species (Table 3) were consistent with the PCA and revealed a limited number of DE genes in brain (four in the hybrid–collared flycatcher (HC) comparison and eight in the hybrid–pied flycatcher (HP) comparison). In line with the clustering of F_1_ hybrids between their parental species, testis also showed a relatively limited number of DE genes in the hybrid/parental species comparisons (61 in each case), much fewer than was observed in heart (210 in HC, 452 in HP), kidney (500, 1539), and liver (577, 1123). The consistently higher number of DE genes in the HP than in the HC comparison for heart, kidney, and liver provides additional evidence for collared-dominant inheritance in these tissues and indicates that gene expression in F_1_ hybrids is more similar to collared flycatcher than to pied flycatcher. Given that we studied male hybrids, which were the result of crosses between male collared flycatcher and female pied flycatcher, the observation is consistent with sex-specific genomic imprinting (Reik and Walter 2001; Gregg et al. 2010). However, existence of genomic imprinting in birds is still under debate (Frésard et al. 2014). Moreover, biased inheritance patterns in F_1_ hybrids toward one of the parental species irrespective of the direction of the cross may primarily reflect dominance relationships between orthologous alleles of gene regulatory elements (McManus et al. 2010; Combes et al. 2015).

**Table 3.** Number of differentially expressed genes between collared flycatcher and pied flycatcher (CP), between F_1_ hybrids and pied flycatcher (HP), and between F_1_ hybrids and collared flycatcher (HC) in five different tissues

Tissue	CP	HP	HC
Brain	52	8	4
Heart	141	452	210
Kidney	485	1539	500
Liver	286	1123	577
Testis	965	61	61

Only genes that could be assigned a chromosomal location are included in the list.

### Distinct mode of gene expression inheritance in testis

Misexpression in F_1_ hybrids is defined by an expression level being either lower or higher in hybrids than in any of the parental species, that is, underdominant or overdominant inheritance of gene expression. We found evidence for abundant hybrid misexpression in heart, kidney, and liver but not in brain and testis (Table 3). For example, there were 1539 DE genes in kidney in the HP comparison but only 485 in the interspecific CP comparison of the same tissue. In contrast, for testis the number of differently expressed genes was vastly higher in the interspecific CP (965) than in the HP (61) or HC (61) comparisons. Again, this suggests that regulatory evolution is highly tissue-specific.

In line with the additive inheritance pattern suggested by the PCA, all testis genes that were differentially expressed in the HC as well as the HP comparisons were also differentially expressed between collared flycatcher and pied flycatcher. They consistently showed intermediate expression levels in the F_1_ hybrids (Supplemental Fig. S4). We thus found no evidence that misexpression of genes in testis could explain the observed sterility of male F_1_ hybrids. Also, misexpression was generally not more pronounced for Z-linked genes than for autosomal genes (Supplemental Table S3).

**Figure 3.**
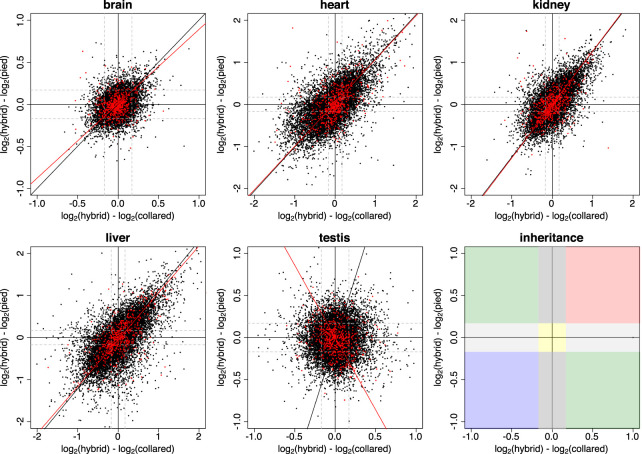
Inheritance mode of gene expression patterns in natural F_1_ hybrids of collared flycatcher and pied flycatcher in five tissues. The scatterplots show shrunken log_2_-fold changes between F_1_ hybrids and collared flycatcher on the *x*-axis, and between F_1_ hybrids and pied flycatcher on the *y*-axis. One dot represents the inheritance pattern of one gene. Autosomal genes are shown in black, genes located on the Z Chromosome are shown in red. The black and the red type II regression lines represent the major variation in log_2_-fold changes for autosomal and Z-linked genes, respectively. Vertical and horizontal black solid lines indicate the coordinate axes. Gray dashed lines indicate the fold-change threshold of 1.125, used for classification of inheritance patterns. *Right*, *bottom* panel: The color code illustrates the thresholds used for classification of genes, with conserved in yellow, pied-dominant inheritance in light gray, collared-dominant inheritance in dark gray, additive inheritance in green, overdominant inheritance in red, and underdominant inheritance in blue.

**Figure 4.**
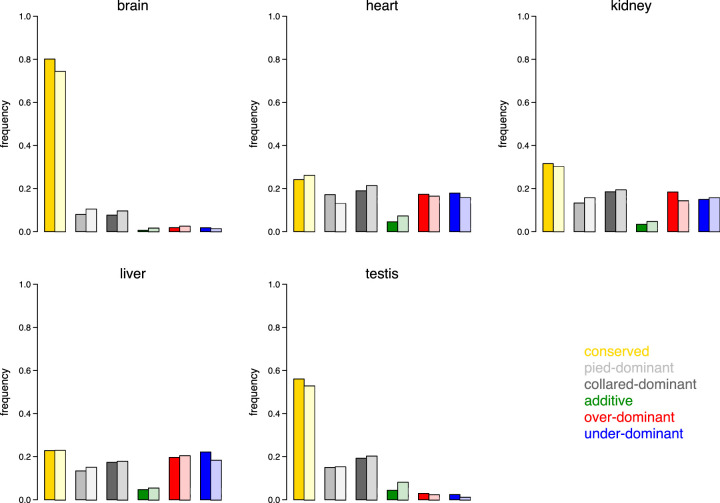
Inheritance mode of gene expression patterns across the five tissues, separately for genes located on autosomes (opaque colors) and the Z Chromosome (transparent colors). The height of the bars shows the frequency of genes in each of the categories.

To study the inheritance patterns of gene expression across tissues in more detail, we investigated the relationship between HC and HP expression differences based on log_2_-fold changes. This approach allowed us to classify genes into six categories: (1) conserved genes; (2) genes showing pied-dominant inheritance; (3) collared-dominant inheritance; (4) additive inheritance; (5) overdominant inheritance; and (6) underdominant inheritance (McManus et al. 2010). Figure 3 shows log_2_-fold changes between pied flycatcher and F_1_ hybrids against log_2_-fold changes between collared flycatcher and F_1_ hybrids across tissues. While brain, heart, kidney, and liver showed a clear trend toward under- and overdominant inheritance for autosomal as well as Z-linked genes (indicated by the type II regression lines), such trend was not observed in the testis. Instead, for testis the regression lines suggested a primary mode of collared-dominant inheritance for autosomal genes and a primary mode of additive inheritance for Z-linked genes. Comparison of the distribution of different modes of inheritance across tissues clearly supports a distinct pattern for gene expression inheritance in testis (Fig. 4). A higher frequency of collared-dominant than pied-dominant inheritance is again consistent with biased inheritance patterns toward the collared flycatcher.

### The transcriptome provides no evidence for differences in testis cell composition between F_1_ hybrids and their parental species

**Figure 5.**
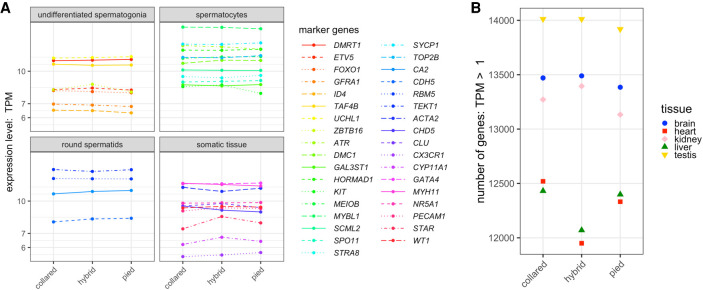
Transcription profile of the testis. (*A*) Gene expression levels (inTPM) of spermatogenesis cell-type marker genes categorized into four different stages—undifferentiated spermatogonia, spermatocytes, round spermatids, and testis niche cells—in testis of collared flycatcher, pied flycatcher, and their F_1_ hybrids. (*B*) Number of genes expressed at an expression level TPM > 1 for the five tissues, for collared flycatcher, pied flycatcher, and their F_1_ hybrids.

We found motile sperm with forward motility in the ejaculates of all 10 collared flycatcher and pied flycatcher males. In contrast, we were not able to detect any sperm cells in the ejaculates of the three F_1_ hybrid flycatcher males, even though their testes were of similar size as compared to the testis of purebred males (Supplemental Fig. S5). Because of this lack of functional sperm production in F_1_ hybrids, it is possible that their testes have a different cell-type composition compared to samples of the parental species due to lower production or absence of sperm precursor cells in the testis (i.e., spermatogonia, spermatocytes, and spermatids). Under such a scenario, we would expect that cell-type markers characteristic of spermatogenesis show lower expression levels in F_1_ hybrids than in the parental species, that is, underdominant inheritance. In addition, misexpression of somatic cells that are essential constituents of the germline stem cell niche (i.e., cells contributing to the regulation of testes stem cell renewal and differentiation) might be expected if they are at the origin of spermatogenesis dysfunction in F_1_ hybrids. Note that misexpression patterns identified by the differential gene expression analysis did not reveal any candidate genes that followed such expression patterns in testis. To corroborate these findings, we specifically investigated expression levels of spermatogenesis cell-type markers (Hermann et al. 2018). This analysis revealed that neither cell-type markers of early spermatogenesis, late spermatogenesis, or testis niche cells show consistently deviating expression levels in testis samples of F_1_ hybrids (Fig. 5A). We therefore find no evidence for differences in cell composition between testes samples of the parental species and their F_1_ hybrids.

A characteristic signature of spermatogenesis is spurious transcription as a result of a permissive chromatin environment during this process (Soumillon et al. 2013). Thus, if spermatogenesis is interrupted at an early stage in the F_1_ hybrids, this should lead to a reduced signature of spurious transcription in the testis samples of F_1_ hybrids. To investigate the signature of spurious transcription during spermatogenesis, we adapted the approach by Soumillon et al. (2013) and counted the number of genes that showed a mean gene expression level of TPM > 1 separately for each of the tissues (Fig. 5B). While for brain, heart, kidney, and liver, the number of expressed genes in F_1_ hybrids either fell below or above the range of the parental species, for testis the number of expressed genes in F_1_ hybrids was within the range of the parental species (collared flycatcher: 14,012 genes, F_1_ hybrids: 14,011 genes, and pied flycatcher: 13,920 genes). This provides further evidence that spermatogenesis takes place in F_1_ hybrid individuals at least until early-stage spermatids are produced and is consistent with earlier findings (Ålund et al. 2013). We therefore find no evidence that potential differences in cell composition between testes of the parental species and their F_1_ hybrids manifest in the transcriptome.

### *cis*-regulatory variation plays a role in the rapid divergence of gene expression in testis

We sought to test the hypothesis that different combinations of *cis*- and *trans*-regulatory changes can explain the distinct mode of gene expression inheritance in testis. Since only 737 genes have fixed coding sequence differences between collared flycatcher and pied flycatcher, we could not use the approach by Wittkopp et al. (2004), designed for crosses of species/inbred lines with substantially divergent genomes, to study regulatory evolution. Instead, we used a protocol suitable for natural populations, where polymorphism is abundant, that tests for an association between differential gene expression and allele-specific expression in F_1_ hybrids as well as ASE in their parental species. ASE was determined using a novel approach that aggregates information across multiple SNPs in a gene and produces individual-based, gene-level tests for ASE (Wang et al. 2018).

Segregating variation in *cis*-regulatory elements forms the basis for ASE in parental species but can also contribute to ASE in F_1_ hybrids. Therefore, a signal of ASE in the parental species and in F_1_ hybrids is not indicative of *cis*-regulatory divergence between species. Only a signal of ASE specific to F_1_ hybrids supports the role of *cis*-regulatory changes in gene expression divergence. We found a statistically significant association between differential gene expression and ASE in both the parental species and in F_1_ hybrids for kidney and liver (Table 4). For testis, differential gene expression and ASE were only associated in F_1_ hybrids. This again indicates tissue-specific patterns of regulatory evolution and a role of *cis*-regulatory sequences in the rapid divergence of gene expression in testis.

**Table 4.** Association between differential gene expression (log_2_-fold changes between collared flycatcher and pied flycatcher) and ASE in either F_1_ hybrids, collared flycatcher, or pied flycatcher across the five tissues for genes located on autosomes

Tissue	F_1_ hybrids	Collared flycatcher	Pied flycatcher
Brain	8.96 × 10^−1^	5.78 × 10^−2^	4.78 × 10^−2 a^
Heart	8.32 × 10^−1^	1.07 × 10^−1^	9.55 × 10^−1^
Kidney	**1.29 × 10^−2^**	**2.39 × 10^−2^**	**2.04 × 10^−2^**
Liver	**1.86 × 10^−2^**	**1.27 × 10^−3^**	1.08 × 10^−1^
Testis	**3.61 × 10^−2^**	1.40 × 10^−1^	8.58 × 10^−1^

The table reports *P*-values based on a Mann–Whitney *U*test between log_2_-fold changes for ASE genes and non-ASE genes. Significant associations (*P*-value < 0.05) are highlighted in bold**.**

^a^The association showed higher log_2_-fold changes for non-ASE genes than for ASE genes and is of the opposite direction than significant associations highlighted in bold.

*cis*-mediated variation could be caused by sequence changes in binding motifs or by epigenetic changes and remodeling of surrounding chromatin. To test if sequence differences in *cis*-regulatory regions between collared flycatcher and pied flycatcher were associated with differential gene expression, we analyzed fixed differences in conserved noncoding elements (CNEs) located either within 5 kb upstream of or in introns of each gene (Table 5). Such elements are prominent candidates for representing *cis*-regulatory regions, such as promoters, enhancers, silencers, etc. (Wittkopp and Kalay 2012; Polychronopoulos et al. 2017; Glaser-Schmitt et al. 2018). Once again, this revealed a testis-specific signal, where genes with fixed CNE differences had significantly higher expression divergence between species specifically in testis compared to genes without fixed CNE differences.

**Table 5.** Association between log_2_-fold changes between collared and pied flycatchers and fixed differences in gene-associated CNEs for genes located on autosomes

Tissue	Brain	Heart	Kidney	Liver	Testis
*P*-value	7.94 × 10^−2^	2.94 × 10^−1^	7.06 × 10^−1^	6.39 × 10^−1^	**7.69 × 10^−3^**

The table reports *P*-values based on a Mann–Whitney *U*test between log_2_-fold changes for genes showing fixed differences in their associated CNEs and genes not showing fixed differences in their associated CNEs. Significant associations (*P*-value < 0.05) are highlighted in bold.

While lack of an association between differential gene expression and fixed CNE differences for brain, heart, kidney, and liver does not rule out the possibility that gene expression in those tissues is controlled by more distal *cis*-regulatory elements, lack of an association between differential gene expression and ASE in F_1_ hybrids for the same tissues is not consistent with this explanation. Alternative explanations could be that expression differences in those tissues are driven by divergence in *trans*-acting factors, or that *cis*-regulatory changes are compensated by changes in relevant *trans*-acting factors and therefore do not lead to differential gene expression. In the latter case, we would expect an overrepresentation of ASE among genes that show conserved expression level between parental species but are misexpressed in F_1_ hybrids. We classified conserved genes into those misexpressed in F_1_ hybrids (i.e., HC and HP) and those not misexpressed in F_1_ (neither HC or HP). There was a significant association between ASE and misexpression in F_1_ hybrids for heart and kidney, and a weak trend in the same direction in liver (Table 6). The signal was specific to ASE in F_1_ hybrids, and not found for ASE in the parental species (see Supplemental Table S4). These results suggest that *cis*-*trans* compensation plays a role in the conservation in gene expression between the parental species and that associated incompatibilities between regulatory elements in F_1_ hybrids contribute to misexpression. In brain and testis, no genes that could be assessed for ASE were classified as misexpressed, and a test for significance could not be performed.

**Table 6.** Association between misexpression and ASE in F_1_ hybrids for genes located on autosomes

	Misexpressed		Not misexpressed		
Tissue	ASE	Non-ASE	ASE	Non-ASE	χ^2^ test
Brain	0	0	177	1959	NA
Heart	5	27	81	1542	**2.25 × 10^−2^**
Kidney	11	40	143	1517	**3.3 × 10^−3^**
Liver	11	64	141	1505	1.07 × 10^−1^
Testis	0	0	209	1763	NA

The table provides numbers of genes showing ASE and non-ASE in F_1_ hybrids in five different organs, separately for misexpressed and not misexpressed genes. Only genes that could be assigned a chromosomal location are included in the list. Differences in the distribution of ASE and non-ASE genes between a misexpressed and not misexpressed gene were assessed by a χ^2^ test. *P*-values are provided in the sixth column. Significant differences (*P*-value < 0.05) are highlighted in bold and underlined if significance was retained after Holm-Bonferroni correction.

## Discussion

Studies of gene expression evolution across a wide range of species suggest that stabilizing selection on gene expression is prevalent and acts to maintain expression levels of genes around their stable optima (Lemos et al. 2005; Gilad et al. 2006; Bedford and Hartl 2009; Chen et al. 2019). However, differences among tissues have been revealed, where reproductive organs belong to the most divergent tissues and brain to the most conserved tissues across species (Khaitovich et al. 2005; Voolstra et al. 2007; Brawand et al. 2011; Sudmant et al. 2015). Furthermore, gene regulation has also been found to be tissue-specific (The GTEx Consortium 2015). Despite these findings, tissue-specific differences have so far mostly been neglected in studies addressing misexpression and Dobzhansky-Muller incompatibilities between divergent regulatory elements in F_1_ hybrids. Abundant misexpression in F_1_ crosses of inbred strains of mice subspecies has been detected in liver (Goncalves et al. 2012) and testis (Mack et al. 2016). In contrast, low levels of misexpression in brain have been observed in crosses of two zebra finch subspecies and were suggested to reflect the slow build-up of postzygotic reproductive isolation observed in many bird lineages (Davidson and Balakrishnan 2016). Our findings suggest that the reported differences in patterns of gene expression evolution might in part be tissue-specific rather than taxon-specific. While brain showed relatively low levels of misexpression in F_1_ hybrids of flycatcher species, we observed abundant misexpression in heart, kidney, and liver.

Epigenetic modifications have been suggested as candidate mechanisms to form the molecular basis of misexpression in hybrids (Groszmann et al. 2013; He et al. 2013). In addition, recent evidence suggests that compensatory divergence of *cis*- and *trans*-regulatory elements in the parental species (be it genetic or epigenetic) can lead to incompatibilities between regulatory elements in hybrids and thereby cause misexpression (Hochholdinger and Hoecker 2007; McManus et al. 2010; Mack and Nachman 2017). Thus, our observation of abundant misexpression in heart, kidney, and liver suggests that compensatory changes are frequent and act to keep gene expression level conserved in those tissues. Stabilizing selection on gene expression may thus cause regulation of gene expression to diverge more rapidly than gene expression itself (Lemos et al. 2005; Gilad et al. 2006; Coolon et al. 2014). However, purifying selection pressure on brain expression patterns seems sufficiently strong to prevent any change in regulatory regions in the first place, keeping the need of compensatory *cis*-*trans* regulatory changes at a low level. Although the exact cause of high selective constraint on gene expression in the brain is not well understood, one hypothesis is that conservation of gene expression is related to severe fitness consequences of changes in gene expression level (Strand et al. 2007; Barbash and Sakmar 2017). It is further possible that we underestimate the abundance of misexpression in F_1_ hybrids since we study adult flycatcher males. If misexpression impacts hybrid survival, results observed for adult birds may not necessarily reflect results observed if younger birds had been sampled.

Testis showed a distinct pattern in flycatchers, with no evidence for misexpression in F_1_ hybrids despite the fact that none of the hybrid individuals studied here showed any evidence for motile sperm cells in their ejaculates. This is in contrast to previous findings of misexpression being more pronounced in sterile than fertile F_1_ hybrids of, for example, *Drosophila* and house mice (Haerty and Singh 2006; Mack et al. 2016), which is suggested to be caused by the accumulation of compensatory *cis*-*trans* regulatory changes. The observed lack of misexpression in flycatcher F_1_ hybrids suggests that compensatory *cis*-*trans* changes do not counteract expression divergence between testis of collared flycatcher and pied flycatcher. Instead, we find evidence for a rapid divergence of gene expression and a role of *cis*-regulatory changes in testis. While we cannot rule out the contribution of *trans*-regulatory changes, such changes are prone to pleiotropic effects whereas complex *cis*-regulatory systems enable the establishment of compartmentalization and a wide diversity of tissue-specific patterns to arise (Arnone and Davidson 1997; Wittkopp et al. 2002; Carroll 2005; Gompel et al. 2005; Saha et al. 2017).

We conclude that the role of tissue-specific regulatory mechanisms may play a more central role for our general understanding of the molecular basis for reproductive isolation and hybrid dysfunction than previously realized. Specifically, our study demonstrates important differences in regulatory evolution among tissues. On the one hand, we find evidence for widespread signatures of misexpression in F_1_ hybrids in heart, kidney, and liver, suggesting a role of incompatibilities between divergent regulatory elements in those tissues. On the other hand, testis showed no signature of misexpression, even though it showed the highest divergence of gene expression among tissues, possibly facilitated by tissue-specific *cis*-regulatory changes. It is therefore unlikely that incompatibilities between *cis*-*trans* regulatory changes explain the observed sterility (lack of normal sperm production) in natural F_1_ hybrids. However, results on testis histology and/or single-cell RNA sequencing data will be necessary to validate this finding and get a better understanding of spermatogenesis in flycatcher F_1_ hybrids.

## Methods

All statistical analyses were completed using R version 3.3.2 (R Core Team 2016).

### Sampling and sample treatment

Thirteen male flycatchers were collected from a population on the Baltic island of Öland (57°10′ N, 16°56′ E) during the breeding season of 2014. These represented five individuals each of collared flycatcher (*F. albicollis*) and pied flycatcher (*F. hypoleuca*), and three first generation (F_1_) hybrids between these species. Classification of species and hybrids was initially done using plumage score (Qvarnström et al. 2010) and subsequently confirmed genetically (further details provided below). As established from banding data, eight of the 13 individuals were in their third calendar year or older, that is, born 2012 or before. Two collared flycatchers, two pied flycatchers, and one hybrid were not banded and their ages are thus unknown.

All studied individuals were held in 2 × 3 × 3 (h × l × w)-meter aviaries containing a nest box, food, water, and nest building material for at least 2 wk prior to tissue sampling. All collared flycatcher and pied flycatcher males were placed with a conspecific female and all F_1_ hybrids with a collared flycatcher female in order to stimulate sperm production. All males were sampled for ejaculates the night before they were sacrificed. Ejaculates were collected through cloacal protuberance massaging (Wolfson 1952) into a tube containing 5 µL of phosphate buffered saline, and 1 µL of this mixture was immediately transferred to a prewarmed (40°C) microscope slide and observed at 100× total magnification using a UB100i microscope fitted with a heated stage and video camera (Projectes i Serveis R + D S.L.). Ejaculates were scanned in a systematic matter for sperm cells showing progressive mobility (Cramer et al. 2016).

### Dissections and storage of tissues

All sacrifices and dissections occurred on the same morning. On the night before tissue collection, all males were transferred to climate chambers with identical conditions for all individuals. All males were sacrificed by decapitation and tissues were collected in this order: blood, brain, heart, lung, gut, liver, right testis, left testis, kidney, and muscle. Brains were subsequently dissected into six regions: hindbrain, midbrain, thalamus, and three telencephalic regions (caudal, rostroventral, and rostrodorsal). Dissections were completed within 20 min for all individuals. Tissue samples were placed in RNAlater in 1.5-mL Eppendorff tubes, snap-frozen in liquid nitrogen, and subsequently stored at −80°C until RNA extraction. All sampling procedures were approved by the Swedish Board of Agriculture (Jordbruksverket – DNR 21-11).

### RNA extraction, library preparation, and sequencing

Tissues were homogenized using a bead beater with ceramic beads (Omni, Intl.), and aliquots of the homogenate were used for total RNA extraction using the Qiagen RNeasy kit (Qiagen) following the manufacturer's instructions. Total RNA for the caudal region of the telencephalon (in this study referred to as brain), heart, kidney, liver, and left testis were used for Illumina paired-end library preparation. RNA integrity numbers were all ≥7.7 (Supplemental Table S5). RNA-seq libraries were prepared from 1 μg total RNA using the TruSeq stranded mRNA sample HT prep kit with poly(A) selection (Illumina, RS-122-2103). Sequencing was performed on an Illumina HiSeq instrument with paired-end reads of 125 bp using v4 chemistry, and a sequencing library for the phage PhiX was included as 1% spike-in in the sequencing run. FastQC was used to check the quality of reads, and no trimming was performed.

### Read mapping

The FicAlb1.5 collared flycatcher assembly (GenBank Accession: GCA_000247815.2) was used as a reference genome, where annotated repeat sequences were masked according to Suh et al. (2018). We mapped RNA-seq reads from each sample to the reference genome using STAR v.2.5.1b (Dobin et al. 2013) with default parameters and the gene feature annotation from Ensembl version 73 (see Uebbing et al. 2016 for detailed description). Gene feature annotation included sequence coordinates for a total of 16,221 annotated flycatcher genes, including 15,561 genes located on autosomes, 623 genes located on the Z Chromosome, and 37 genes located on the mitochondria. To avoid any mapping bias toward the collared flycatcher, we masked all positions in the reference genome that show fixed differences between collared flycatcher and pied flycatcher using SAMtools v.1.3 prior to the mapping (Li et al. 2009). Fixed differences were identified based on polymorphism data for 19 collared and 19 pied flycatchers from the island of Öland retrieved from Burri et al. (2015). Only RNA-seq reads that mapped uniquely to annotated genes were used for further analyses (Supplemental Table S1). After mapping, all duplicate reads were marked and reduced to a single copy using Picard v 2.0.1.

### Genetic classification of species and hybrids

To genetically confirm the identity of all individuals (i.e., whether they were collared flycatchers, pied flycatchers, or F_1_ hybrids), we called SNPs individually and compared them to the fixed differences between collared flycatcher and pied flycatcher described above. More specifically, we pooled uniquely mapped reads from different tissues of the same individual for each species. Then, we called SNPs individually using GATK 3.5.0 following the best practice protocol with recommended parameter settings (Van der Auwera et al. 2013).

To identify the direction of the cross between collared and pied flycatchers, we conducted a phylogenetic analysis based on mitochondrial RNA (mtRNA). We mapped RNA-seq reads from all three hybrid individuals to the mtDNA reference of collared flycatcher assembly FicAlb1.5 and the mtDNA annotation of Ensembl version 73 by using STAR v.2.5.1b with the same parameter settings as described above. We extracted reads that were uniquely mapped to the 37 annotated mitochondrial genes for further analysis. For each hybrid individual, we used GATK version 3.5.0 with default parameter settings to call SNPs. For each individual, we constructed mtDNA sequences by replacing sites on the mtDNA reference with the called nonreference genotype using SAMtools v.1.5. We aligned mtDNA sequences from all individuals and constructed a phylogeny using the maximum parsimony method available in using Seaview version 4 with default parameter settings (Gouy et al. 2010).

### Read counting and differential gene expression analysis

We computed differential gene expression patterns using the DESeq2 package provided in R (Love et al. 2014). Since DESeq2 requires raw read counts as input data, we used HTSeq v0.6.1 (Anders et al. 2015) to generate the input matrix for DESeq2. We limited the read counting to reads with a mapping quality of at least 30. Read counting was configured to handle reverse-stranded sequencing data, and the parameter that controls for overlapping gene features was set to union. For all other parameters in HTSeq, we kept default parameter values. Averages of total counts per sample are provided in Supplemental Table S6.

For each tissue, the count data of all 13 individuals was formatted as an R matrix and used as input data for DESeq2. Differential gene expression analysis was performed for each tissue separately based on pair-wise contrasts. Genes with a FDR adjusted *P*-value < 0.05 were considered to show significant differential gene expression (DE genes). Genes with a FDR adjusted *P*-value ≥ 0.05 were considered to show no differential gene expression (nDE genes). Genes for which differential gene expression could not be assessed were not classified. We further calculated shrunken log_2_-fold changes, which removes the noise associated with log_2_-fold changes for genes with low count values. In order to investigate clustering of individual gene expression patterns in each tissue, we applied a regularized log-transformation on the count data as implemented in DESeq2 and subsequently performed PCA as implemented in the ade4 package (Dray and Dufour 2007). In addition, data matrices were combined across tissues in order to perform principal component analysis (PCA) across tissues and species together. The proportion of variance associated with species and tissue was computed based on between-groups PCA. Statistical significance was evaluated by a Monte-Carlo test on the percentage of explained inertia based on 99 permutations.

### Allele-specific expression analyses

Since we retrieved five individuals for collared flycatcher and pied flycatcher but only three for their hybrids, we only involved three individuals of each parental species in the ASE analyses to balance the power (COL01, COL02, and COL03 for collared flycatcher; PIE01, PIE02, and PIE03 for pied flycatcher). To avoid mapping bias originating from mapping of heterozygous alleles to a single reference genome, we masked SNP positions in the reference genome and repeated the mapping following the same protocol as described above. To be consistent with the differential gene expression analyses, we only considered reads that passed filtering criteria from HTSeq with a mapping quality of at least 30. For each sample, we mapped the filtered reads to the corresponding individual reference genome using STAR v.2.5.1b and obtained allelic counts using SAMtools v.1.5. Additionally, for each sample allelic counts from SNPs that showed evidence of strong mapping bias were removed following Degner et al. (2009).

We used RPASE to conduct individual-based ASE analyses (Wang et al. 2018). To be included in the ASE analysis, we required each gene to contain at least two phased SNPs and each SNP to have minimum coverage of 10 and minimum allelic depth greater than 2. Gene-specific *P*-values were adjusted for multiple testing using Benjamini-Hochberg correction. The number of genes that could be studied for ASE in each tissue and individual is shown in Supplemental Table S7. Since RPASE identifies ASE at the individual level and we are interested in ASE at the population level, we applied further criteria to leverage individual-level ASE up to population-level ASE. To do so, we required genes to have at least two individuals tested for ASE (i.e., we removed genes that had only one individual tested for ASE) and defined genes to show ASE as those that had adjusted *P*-value < 0.05 in at least two individuals.

### Tissue specificity of gene expression

To estimate normalized expression levels in transcripts per million (TPM), we used uniquely mapped reads from STAR and calculated TPM using RSEM 1.2.29 (Li and Dewey 2011) for all 16,221 annotated genes and for each tissue separately. We then calculated tissue specificity following Yanai et al. (2005). Tissue specificity was highly correlated between collared flycatcher, pied flycatcher, and F_1_ hybrids (*r* ≥ 0.97). We used PCA and computed the major variation in tissue specificity across species as a representative of flycatcher-specific tissue specificity.

### Computation of population genomic parameters

Fixed differences between collared flycatcher and pied flycatcher were restricted to the set of sites located in conserved noncoding elements obtained from Craig et al. (2018). We associated CNEs with a gene by considering all CNEs that were located either 5 kb upstream of or in introns of genes as gene-associated CNEs.

We calculated *F*_ST_ based on the filtered polymorphism data from Burri et al. (2015), described above. *F*_ST_ was calculated for 50-kb windows all along the genome using VCFtools v0.1.14 with default settings (Danecek et al. 2011). Moreover, nonsynonymous/synonymous nucleotide diversity (π_N_/π_S_) was calculated based on the site frequency spectrum of zero- and fourfold degenerated sites following Bolívar et al. (2018)_._

### Regression analysis

We investigated the association between differential gene expression and genomic and gene-specific features using a generalized linear regression with binomial error distribution. For this purpose, differential gene expression between collared flycatcher and pied flycatcher was encoded by 1 if the FDR adjusted *P*-value < 0.05, and 0 otherwise. We included a total of six explanatory variables: tissue specificity index τ, gene-wise dispersion parameter ϕ from DESeq2 (Love et al. 2014) as a proxy for the degree of intra-specific variation in gene expression, the number of protein–protein interactions retrieved from Uebbing et al. (2015), flycatcher-specific nonsynonymous/synonymous nucleotide divergence (*d*_N_/*d*_S_) obtained from Bolívar et al. (2016), nonsynonymous/synonymous nucleotide diversity (π_N_/π_S_), and *F*_ST_. In addition, we used log_2_-fold change in gene expression as a response variable in order to check for consistency between results based on 0/1 encoding of differential expression judged by significance and log_2_-fold changes in gene expression. For this purpose, we performed multiple linear regression analyses against the same six explanatory variables.

### Gene expression patterns of spermatogenesis cell-type markers

Cell-type markers for three different stages of spermatogenesis (undifferentiated spermatogonia, spermatocytes, round spermatids) and markers of the germline stem cell niche were inferred from human (Hermann et al. 2018). Gene names were associated with the respective collared flycatcher Ensembl gene ID with BioMart, Ensembl release 98. For each gene that was associated with a unique Ensembl gene ID, mean expression level (in TPM) across testis samples was computed separately for collared flycatcher, pied flycatcher, and F_1_ hybrid individuals.

## Data access

The RNA-seq data generated in this study have been submitted to the NCBI BioProject database (https://www.ncbi.nlm.nih.gov/bioproject/) under accession number PRJNA551584.

## Competing interest statement

The authors declare no competing interests.

## Supplementary Material

Supplemental Material
