# Supplementary material for: Tissue-specific patterns of regulatory changes underlying gene expression differences among Ficedula flycatchers and their naturally occurring F1 hybrids
Source: Genome Res. 2020 Dec;30(12):1727–39. doi: 10.1101/gr.254508.119 (PMC7706733; doi:10.1101/gr.254508.119)
Supplement: Supplemental Material [file supp_30_12_1727__index.html]

Tissue-specific patterns of regulatory changes underlying gene expression differences among Ficedula flycatchers and their naturally occurring F1 hybrids — Tissue-specific patterns of regulatory changes underlying gene expression differences among Ficedula flycatchers and their naturally occurring F1 hybrids — Supplemental Material 

# Tissue-specific patterns of regulatory changes underlying gene expression differences among *Ficedula* flycatchers and their naturally occurring F1 hybrids

## Supplemental Material

- Supplemental\_Material.pdf
