# Supplementary material for: Tissue-specific patterns of regulatory changes underlying gene expression differences among Ficedula flycatchers and their naturally occurring F1 hybrids
Source: Genome Res. 2020 Dec;30(12):1727–39. doi: 10.1101/gr.254508.119 (PMC7706733; doi:10.1101/gr.254508.119)
Supplement: Supplemental Material [file supp_gr.254508.119_Supplemental_Material.pdf]

# Supplemental Material

## Tissue-specific patterns of regulatory changes underlying gene expression differences among *Ficedula* flycatchers and their naturally-occurring $F_1$ hybrids

Carina F. Mugal*, Mi Wang, Niclas Backström, David Wheatcroft, Murielle Ålund, Marie Sémon, S. Eryn McFarlane, Ludovic Dutoit, Anna Qvarnström, and Hans Ellegren

Carina Farah Mugal; email: carina.mugal@ebc.uu.se

Department of Ecology and Genetics
Uppsala University
Norbyvägen 18D
752 36 Uppsala
Sweden

## Table of Contents

Supplemental Analysis ………………………………………………………………………………………... 2 – 4
Supplemental Tables ……………………………………………………………………………………… 5 – 15
Supplemental Figures ……………………………………………………………………………………. 16 – 20

## Supplemental Analysis

### Power analysis

We assessed the power of our differential gene expression analysis based on the DESeq2 package (Love, et al. 2014) with a simulation-based method, PROPER (Wu, et al. 2015). The power of the analysis was estimated separately for each tissue using the same DE thresholds as in the main analysis, i.e. genes with a FDR adjusted *p*-value < 0.05 were considered to show significant differential gene expression (DE genes). We used empirical values taken from the corresponding tissue in the flycatcher dataset for baseline expression levels and biological variation (dispersion). For the simulations, the percentage of DE genes was set to 5%, an approximation based on observed values. We simulated 3 scenarios, 1) a dataset with 5 samples for two species (as in our collared flycatcher, C, and pied flycatcher, P, comparison), 2) a dataset with 3 samples for one species and 5 samples for the other species (as in our $F_1$ hybrid, H, and one parental species, C or P, comparison), and 3) a dataset with 3 samples for one species and 5 samples for two other species (as in comparing 3 $F_1$ hybrids, H, and both parental species, C and P). In order to simulate scenario 3, the PROPER software package was slightly modified (code available upon request). 20 independent simulations were performed for each tissue, and for each scenario. Power values were stratified by deciles of the baseline expression levels.

The power analysis revealed that the power of the differential gene expression analysis was of comparable strength among tissues and scenarios. We found no evidence of systematic differences among tissues and scenarios. Power was lowest for heart, with a median power of 0.62, 0.68, and 0.71 for scenarios 1 – 3, respectively, and highest for testis, with a median power of 0.76, 0.79, and 0.82 for scenarios 1 – 3, respectively. Power of the three other tissues fell in between. Power of all five tissues and scenarios is shown in

Supplemental Figure S2. We therefore find no evidence that lack of power in a particular tissue and/or scenario could explain the findings presented in the main text.

However, we acknowledge that there are some limitations in the power of our differential gene expression study, which is a common problematic in RNA-seq studies of natural populations (Todd, et al. 2016). In order to explore if our study shows comparable power to other recent RNA-seq studies of natural populations, we investigated the power of a RNA-seq study in three primates based on a sample size of 4 for each species (Blake, et al. 2020). We investigated four different scenarios, 1) a dataset with 3 samples for one species and 5 samples for the other species (as in our $F_1$ hybrid, H, and one parental species, C or P, comparison), 2) a dataset with 5 samples for two species (as in our collared flycatcher, C, and pied flycatcher, P, comparison), 3) a dataset with 3 samples for one species and 5 samples for two other species (as in comparing 3 $F_1$ hybrids, H, and both parental species, C and P), and 4) a dataset with 4 samples for two species (as in Blake et al. 2020). Power of all four tissues investigated in the primate study for the four different scenarios is shown in Supplemental Figure S3.

Comparison of the power of the differential gene expression analysis presented in our study with that in Blake et al. 2020 shows that the power is similar in the two studies. If anything, there is a slightly higher power in our study. Power of Blake et al. 2020 was lowest for heart, with a median power of 0.57, followed by liver (0.58), lung (0.64) and kidney (0.67).

Also, note that we not only performed differential gene expression analysis, but also corroborated the findings by investigating inheritance patterns based on $log_2$-fold changes (see Figures 3 and 4 of the main text). The latter analysis doesn't depend on significance thresholds but instead investigates trends. Since results were consistent between differential gene expression analysis and

trends of inheritance patterns based on $\log_2$-fold changes, this provides further support that results presented in the main text are robust.

**References**

Blake LE, Roux J, Hernando-Herraez I, Banovich NE, Perez RG, Hsiao CJ, Eres I, Cuevas C, Marques-Bonet T, Gilad Y. 2020. A comparison of gene expression and DNA methylation patterns across tissues and species. *Genome Res* 30:250-262.

Love MI, Huber W, Anders S. 2014. Moderated estimation of fold change and dispersion for RNA-seq data with DESeq2. *Genome Biol* 15:550.

Todd EV, Black MA, Gemmell NJ. 2016. The power and promise of RNA-seq in ecology and evolution. *Mol Ecol* 25:1224-1241.

Wu H, Wang C, Wu Z. 2015. PROPER: comprehensive power evaluation for differential expression using RNA-seq. *Bioinformatics* 31:233-241.

Supplemental Table S1: Summary of RNA-seq read statistics, (A) collared flycatcher, (B) pied flycatcher, and (C) $F_1$ hybrids.

(A)

| | Number of raw reads [$10^{07}$] / Uniquely mapped reads % | | | | | |
|---|---|---|---|---|---|---|
| | brain | heart | kidney | liver | testis | Mean per ind |
| COL01 | 4.46 / 54.54% | 5.11 / 42.14% | 5.22 / 60.44% | 4.77 / 63.40% | 5.09 / 71.48% | 4.93 / 58.4% |
| COL02 | 3.81 / 61.88% | 5.16 / 46.64% | 4.81 / 63.59% | 4.96 / 55.79% | 4.76 / 60.67% | 4.70 / 57.71% |
| COL03 | 4.59 / 59.13% | 5.08 / 47.67% | 5.10 / 59.58% | 5.23 / 49.41% | 4.42 / 68.51% | 4.88 / 56.86% |
| COL04 | 4.61 / 67.32% | 5.49 / 48.73% | 4.83 / 51.19% | 5.30 / 65.05% | 5.07 / 73.72% | 5.06 / 61.2% |
| COL05 | 4.72 / 71.44% | 4.82 / 48.07% | 5.04 / 59.78% | 4.92 / 61.90% | 4.67 / 69.34% | 4.83·v / 62.11% |
| Mean per tissue | 4.44 / 62.86% | 5.13 / 46.65% | 5.00 / 58.92% | 5.04 / 59.11% | 4.80 / 68.74% | |

(B)

| | Number of raw reads [$10^{07}$] / Uniquely mapped reads % | | | | | |
|---|---|---|---|---|---|---|
| | brain | heart | kidney | liver | testis | Mean per ind |
| PIE01 | 3.39 / 62.85% | 4.02 / 42.54% | 3.68 / 54.00% | 4.02 / 55.14% | 3.39 / 64.58% | 3.70 / 55.82% |
| PIE02 | 3.94 / 59.01% | 4.06 / 51.46% | 4.26 / 61.13% | 4.29 / 65.52% | 3.98 / 64.94% | 4.11 / 60.41% |
| PIE03 | 3.85 / 59.48% | 4.63 / 49.79% | 4.68 / 59.83% | 4.34 / 52.26% | 4.10 / 62.55% | 4.32 / 56.78% |
| PIE04 | 3.39 / 62.12% | 4.59 / 49.99% | 4.20 / 60.13% | 4.47 / 57.82% | 4.48 / 71.37% | 4.22 / 60.29% |
| PIE05 | 4.35 / 64.09% | 3.49 / 52.20% | 4.75 / 53.89% | 4.55 / 40.60% | 4.86 / 64.72% | 4.40 / 55.1% |
| Mean per tissue | 3.78 / 61.51% | 4.16 / 49.2% | 4.31 / 57.8% | 4.34 / 54.27% | 4.16 / 65.63% | |

(C)

| | Number of raw reads [$10^{07}$] / Uniquely mapped reads % | | | | | |
|---|---|---|---|---|---|---|
| | brain | heart | kidney | liver | testis | Mean per ind |
| HYB01 | 4.44 / 65.20% | 4.23 / 41.82% | 3.96 / 59.54% | 4.04 / 62.08% | 4.24 / 69.46% | 4.18 / 59.62% |
| HYB02 | 4.35 / 69.30% | 4.52 / 45.30% | 4.15 / 59.84% | 4.69 / 56.11% | 4.13 / 72.44% | 4.37 / 60.6% |
| HYB05 | 4.09 / 65.43% | 3.74 / 43.94% | 5.79 / 55.00% | 3.90 / 57.22% | 4.46 / 70.44% | 4.40 / 58.41% |
| Mean per tissue | 4.29 / 66.64% | 4.16 / 43.69% | 4.63 / 58.13% | 4.21 / 58.47% | 4.28 / 70.78% | |

Supplemental Table S2: Multiple linear regression (MLR) analysis of $log_2$-fold in gene expression between collared flycatcher and pied flycatcher against the number of protein-protein interactions (PPI), tissue-specificity ($\tau$), intraspecies variation in gene expression ($\phi$), the ratio of non-synonymous to synonymous diversity ($\pi_N/\pi_S$) and divergence ($d_N/d_S$) and genomic differentiation between collared flycatcher and pied flycatcher ($F_{ST}$) as candidate explanatory variables. For each tissue, the *t*-statistic of the association and *p*-values are reported. Significant associations (*p*-value < 0.001) are highlighted in bold.

| | brain | | heart | | kidney | | liver | | testis | |
|---|---|---|---|---|---|---|---|---|---|---|
| | *t*-stat. | *p*-value | *t*-stat. | *p*-value | *t*-stat. | *p*-value | *t*-stat. | *p*-value | *t*-stat. | *p*-value |
| PPI | -1.80 | $7.13\times10^{-02}$ | -1.96 | $4.96\times10^{-02}$ | -1.49 | $1.37\times10^{-01}$ | -2.69 | $7.08\times10^{-03}$ | -2.41 | $1.61\times10^{-02}$ |
| $\tau$ | 5.41 | **$6.53\times10^{-08}$** | 20.82 | **$3.32\times10^{-93}$** | 19.00 | **$2.51\times10^{-78}$** | 19.39 | **$2.01\times10^{-81}$** | 14.42 | **$1.90\times10^{-46}$** |
| $\phi$ | -14.33 | **$6.94\times10^{-46}$** | -20.14 | **$1.72\times10^{-87}$** | -17.02 | **$1.45\times10^{-63}$** | -21.11 | **$1.29\times10^{-95}$** | -12.84 | **$2.84\times10^{-37}$** |
| $\pi_N/\pi_S$ | 0.64 | $5.20\times10^{-01}$ | 0.20 | $8.44\times10^{-01}$ | 0.48 | $6.31\times10^{-01}$ | 0.68 | $4.99\times10^{-01}$ | 0.74 | $4.60\times10^{-01}$ |
| $d_N/d_S$ | 8.01 | **$1.31\times10^{-15}$** | 3.82 | **$1.35\times10^{-04}$** | 0.63 | $5.28\times10^{-01}$ | 5.53 | **$3.30\times10^{-08}$** | 0.62 | $5.35\times10^{-01}$ |
| $F_{ST}$ | 1.85 | $6.49\times10^{-02}$ | 0.88 | $3.80\times10^{-01}$ | 2.08 | $3.77\times10^{-02}$ | 2.76 | $5.80\times10^{-03}$ | 3.59 | **$3.31\times10^{-04}$** |

Supplemental Table S3: Numbers of differentially expressed (DE) genes and non-differentially (nDE) expressed genes between in five different tissues, separately for autosomes and the Z chromosome (A) for the collared flycatcher and $F_1$ hybrid comparison, and (B) for the pied flycatcher and $F_1$ hybrid comparison. Only genes that could be assigned a chromosomal location are included in the list. Differences in the distribution of DE and nDE genes between autosomes and the Z chromosome were assessed by a $\chi^2$-test Significant differences (*p*-value < 0.05) are highlighted in bold.

(A)

| | Autosomes | | Z chromosome | | |
|---|---|---|---|---|---|
| tissue | DE | nDE | DE | nDE | *p*-value |
| brain | 4 | 13405 | 0 | 588 | 1.0 |
| heart | 198 | 10327 | 12 | 451 | $2.9\times10^{-01}$ |
| kidney | 478 | 10917 | 22 | 482 | $9.0\times10^{-01}$ |
| liver | 549 | 9611 | 28 | 415 | $4.7\times10^{-01}$ |
| testis | 55 | 11306 | 6 | 498 | **$4.9\times10^{-02}$** |

(B)

| | Autosomes | | Z chromosome | | |
|---|---|---|---|---|---|
| tissue | DE | nDE | DE | nDE | *p*-value |
| brain | 8 | 12003 | 0 | 522 | 1.0 |
| heart | 431 | 10094 | 21 | 442 | $7.1\times10^{-01}$ |
| kidney | 1474 | 10149 | 65 | 448 | 1.0 |
| liver | 1074 | 9086 | 49 | 394 | $7.5\times10^{-01}$ |
| testis | 58 | 11303 | 3 | 501 | 1.0 |

Supplemental Table S4: Association between mis-expression and ASE in (A) collared flycatcher and (B) pied flycatcher for genes located on autosomes. The table provides numbers of genes showing ASE and nonASE in five different organs, separately for mis-expressed and not mis-expressed genes. Only genes that could be assigned a chromosomal location are included in the list. Differences in the distribution of ASE and nonASE genes between a mis-expressed and not mis-expressed genes were assessed by a $\chi^2$-Test. *P*-values are provided in the sixth column. Significant differences ($p$-value < 0.05) are highlighted in bold.

(A)

| | mis-expressed | | not mis-expressed | | |
|---|---|---|---|---|---|
| tissue | ASE | nonASE | ASE | nonASE | $\chi^2$-Test |
| brain | 0 | 0 | 160 | 1976 | NA |
| heart | 2 | 30 | 150 | 1473 | $7.86\times10^{-01}$ |
| kidney | 7 | 44 | 149 | 1511 | $3.61\times10^{-01}$ |
| liver | 10 | 65 | 215 | 1431 | 1.0 |
| testis | 0 | 0 | 197 | 1775 | NA |

(B)

| | mis-expressed | | not mis-expressed | | |
|---|---|---|---|---|---|
| tissue | ASE | nonASE | ASE | nonASE | $\chi^2$-Test |
| brain | 0 | 0 | 162 | 1974 | NA |
| heart | 4 | 28 | 112 | 1511 | $3.79\times10^{-01}$ |
| kidney | 5 | 46 | 132 | 1528 | $8.27\times10^{-01}$ |
| liver | 11 | 64 | 195 | 1451 | $5.80\times10^{-01}$ |
| testis | 0 | 0 | 180 | 1792 | NA |

Supplemental Table S5: Summary of RNA integrity numbers for collared flycatcher, pied flycatcher, and $F_1$ hybrid samples.

| | **brain** | **heart** | **kidney** | **liver** | **testis** |
|---|---|---|---|---|---|
| **COL01** | 9.0 | 8.2 | 9.6 | 8.6 | 9.4 |
| **COL02** | 9.3 | 8.8 | 9.7 | 9.7 | 9.5 |
| **COL03** | 9.2 | 7.9 | 9.7 | 8.8 | 9.4 |
| **COL04** | 9.1 | 7.8 | 8.5 | 8.8 | 9.6 |
| **COL05** | 9.3 | 8.5 | 9.6 | 9.5 | 9.5 |
| **PIE01** | 9.4 | 8.9 | 9.6 | 7.7 | 9.6 |
| **PIE02** | 9.3 | 8.8 | 9.8 | 8.9 | 9.5 |
| **PIE03** | 9.4 | 8.9 | 9.8 | 9.5 | 9.3 |
| **PIE04** | 9.2 | 8.8 | 9.2 | 9.6 | 9.3 |
| **PIE05** | 8.8 | 8.9 | 9.7 | 8.8 | 9.6 |
| **HYB01** | 9.3 | 9.0 | 9.5 | 9.2 | 9.5 |
| **HYB02** | 9.2 | 8.9 | 9.9 | 8.7 | 9.4 |
| **HYB05** | 9.4 | 8.6 | 9.8 | 9.2 | 9.6 |

Supplemental Table S6: Summary of total counts from HTseq for (A) collared flycatcher, (B) pied flycatcher, and (C) $F_1$ hybrids.

(A)

| | Number of total counts [$10^{07}$] / percentage of counts relative to the number of raw reads | | | | | |
|---|---|---|---|---|---|---|
| | brain | heart | kidney | liver | testis | Mean per ind |
| COL01 | 1.26 / 28.27% | 1.35 / 26.37% | 2.59 / 49.65% | 2.91 / 61.02% | 2.76 / 54.32% | 2.18 / 43.93% |
| COL02 | 1.30 / 34.07% | 1.65 / 31.92% | 2.51 / 52.14% | 3.04 / 61.32% | 2.20 / 46.14% | 2.14 / 45.12% |
| COL03 | 1.60 / 34.87% | 1.62 / 31.92% | 2.43 / 47.63% | 2.37 / 45.33% | 2.37 / 53.62% | 2.08 / 42.68% |
| COL04 | 1.67 / 36.29% | 2.03 / 36.97% | 1.81 / 37.52% | 3.41 / 64.36% | 2.75 / 54.35% | 2.34 / 45.9% |
| COL05 | 1.64 / 34.7% | 1.89 / 39.13% | 2.49 / 49.46% | 3.11 / 63.3% | 2.32 / 49.64% | 2.29 / 47.24% |
| Mean per tissue | 1.49 / 33.64% | 1.71 / 33.26% | 2.37 / 47.28% | 2.97 / 59.07% | 2.48 / 51.62% | |

(B)

| | Number of total counts [$10^{07}$] / percentage of counts relative to the number of raw reads | | | | | |
|---|---|---|---|---|---|---|
| | brain | heart | kidney | liver | testis | Mean per ind |
| PIE01 | 1.11 / 32.77% | 1.30 / 32.34% | 1.50 / 40.88% | 2.00 / 49.82% | 1.76 / 52.05% | 1.54 / 41.57% |
| PIE02 | 1.29 / 32.83% | 1.58 / 38.84% | 2.20 / 51.64% | 2.79 / 65.01% | 1.98 / 49.61% | 1.97 / 47.59% |
| PIE03 | 1.18 / 30.69% | 1.81 / 38.99% | 2.19 / 46.89% | 2.03 / 46.7% | 1.89 / 46.15% | 1.82 / 41.88% |
| PIE04 | 0.99 / 29.17% | 1.80 / 39.33% | 1.99 / 47.46% | 2.67 / 59.68% | 2.54 / 56.72% | 2.00 / 46.47% |
| PIE05 | 1.55 / 35.54% | 1.34 / 38.44% | 2.06 / 43.37% | 1.48 / 32.59% | 2.60 / 53.55% | 1.81 / 40.7% |
| Mean per tissue | 1.22 / 32.2% | 1.57 / 37.59% | 1.99 / 46.05% | 2.19 / 50.76% | 2.15 / 51.62% | |

(C)

| | Number of total counts [$10^{07}$] / percentage of counts relative to the number of raw reads | | | | | |
|---|---|---|---|---|---|---|
| | brain | heart | kidney | liver | testis | Mean per ind |
| HYB01 | 1.54 / 34.65% | 1.04 / 24.69% | 1.96 / 49.6% | 2.67 / 66.14% | 2.25 / 52.97% | 1.89 / 45.61% |
| HYB02 | 1.62 / 37.26% | 1.60 / 35.47% | 2.08 / 50.04% | 2.26 / 48.28% | 2.16 / 52.33% | 1.94 / 44.68% |
| HYB05 | 1.33 / 32.44% | 1.25 / 33.46% | 2.63 / 45.42% | 1.92 / 49.32% | 2.50 / 56.11% | 1.93 / 43.35% |
| Mean per tissue | 1.49 / 34.78% | 1.30 / 31.21% | 2.22 / 48.35% | 2.29 / 54.58% | 2.30 / 53.8% | 0 |

Supplemental Table S7: Number of genes that were tested for ASE in each tissue and individual for collared flycatcher, pied flycatcher, and their $F_1$ hybrids.

| | brain | heart | kidney | liver | testis |
|---|---|---|---|---|---|
| COL01 | 2183 | 1731 | 2219 | 1768 | 2385 |
| COL02 | 2342 | 1741 | 2274 | 1910 | 2434 |
| COL03 | 2440 | 2025 | 2298 | 1882 | 2394 |
| PIE01 | 1940 | 1434 | 1867 | 1572 | 1958 |
| PIE02 | 1942 | 1516 | 2033 | 1736 | 2094 |
| PIE03 | 2156 | 1664 | 2106 | 1710 | 2236 |
| HYB01 | 2901 | 1688 | 2754 | 2075 | 2920 |
| HYB02 | 3050 | 2092 | 2687 | 2328 | 2989 |
| HYB05 | 2909 | 2029 | 2892 | 2386 | 2956 |

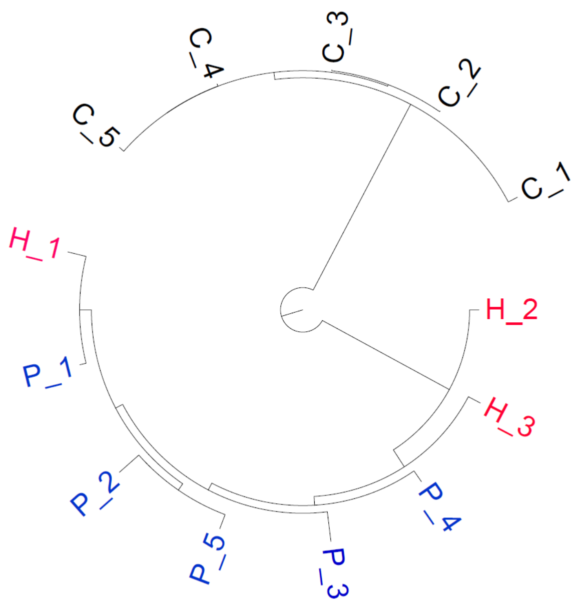


Supplemental Figure S1: Phylogenetic tree of mtDNA sequences of genes expressed in kidney, of 5 collared flycatcher individuals (black), 5 pied flycatcher individuals (blue), and 3 $F_1$ hybrids (red).

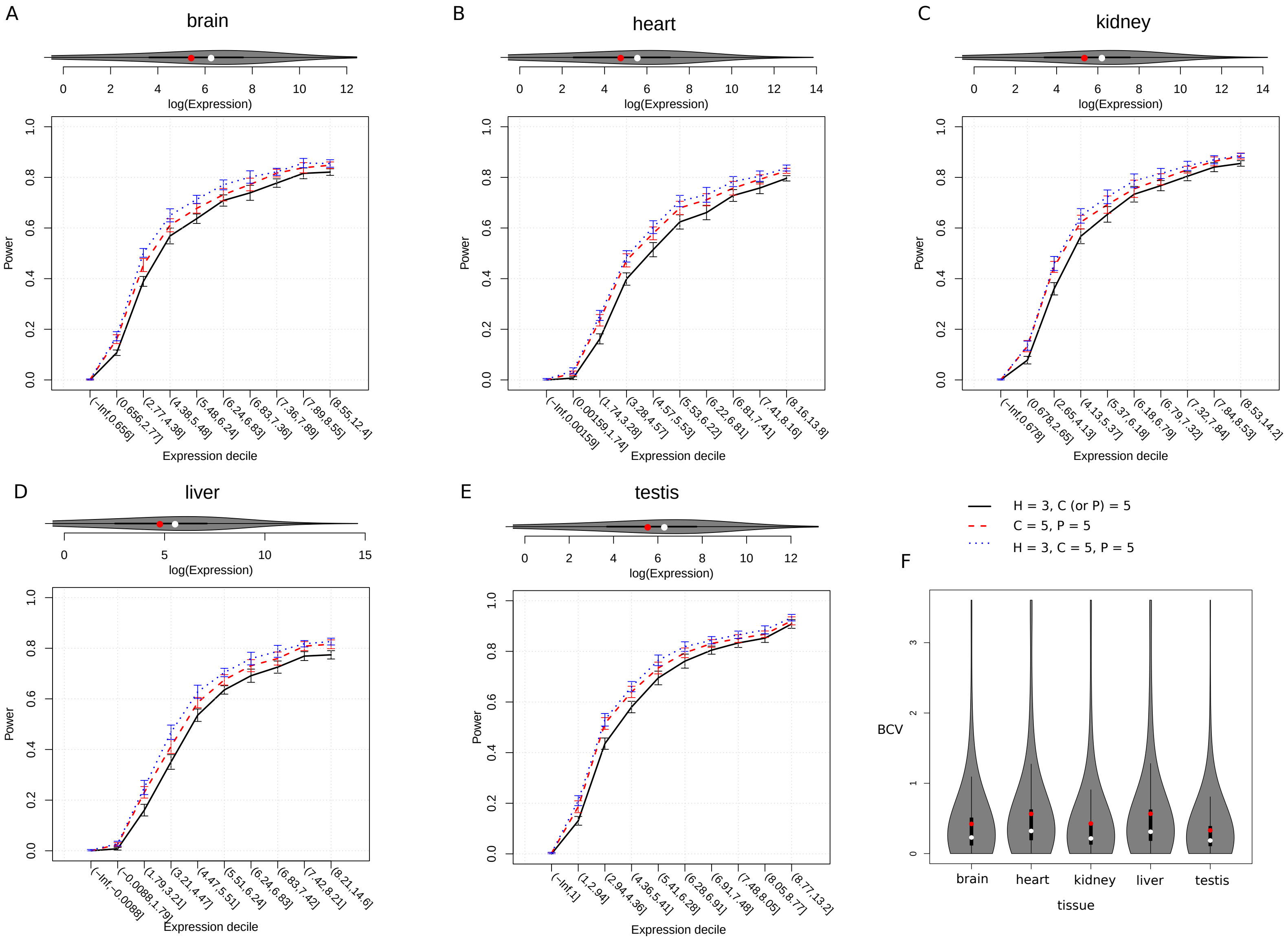
A
brain
B
heart
C
kidney
D
liver
E
testis
F
log(Expression)
Power
Expression decile
H = 3, C (or P) = 5
C = 5, P = 5
H = 3, C = 5, P = 5
BCV
brain
heart
kidney
liver
testis
tissue

Supplemental Figure S2: Power of our differential gene expression analysis based on DeSeq2 as a function of expression level, separately for each tissue. A – E: Power values are stratified by deciles of the baseline expression levels. Black solid lines represent a scenario comparing 3 $F_1$ hybrid samples (H) and 5 pure species samples (C or P). Red dashed lines represent a scenario comparing two species based on 5 samples each (C and P). Blue dotted lines represent a scenario comparing 3 $F_1$ hybrid samples (H) and two parental species based on 5 samples each (C and P). Violin plots directly above each panel represent the distribution of expression levels for each tissue, with median values in white and mean values in red. F: Violin plots represent the distribution of the biological coefficient of variation (BCV) separately for each tissue, with median values in white and mean values in red.

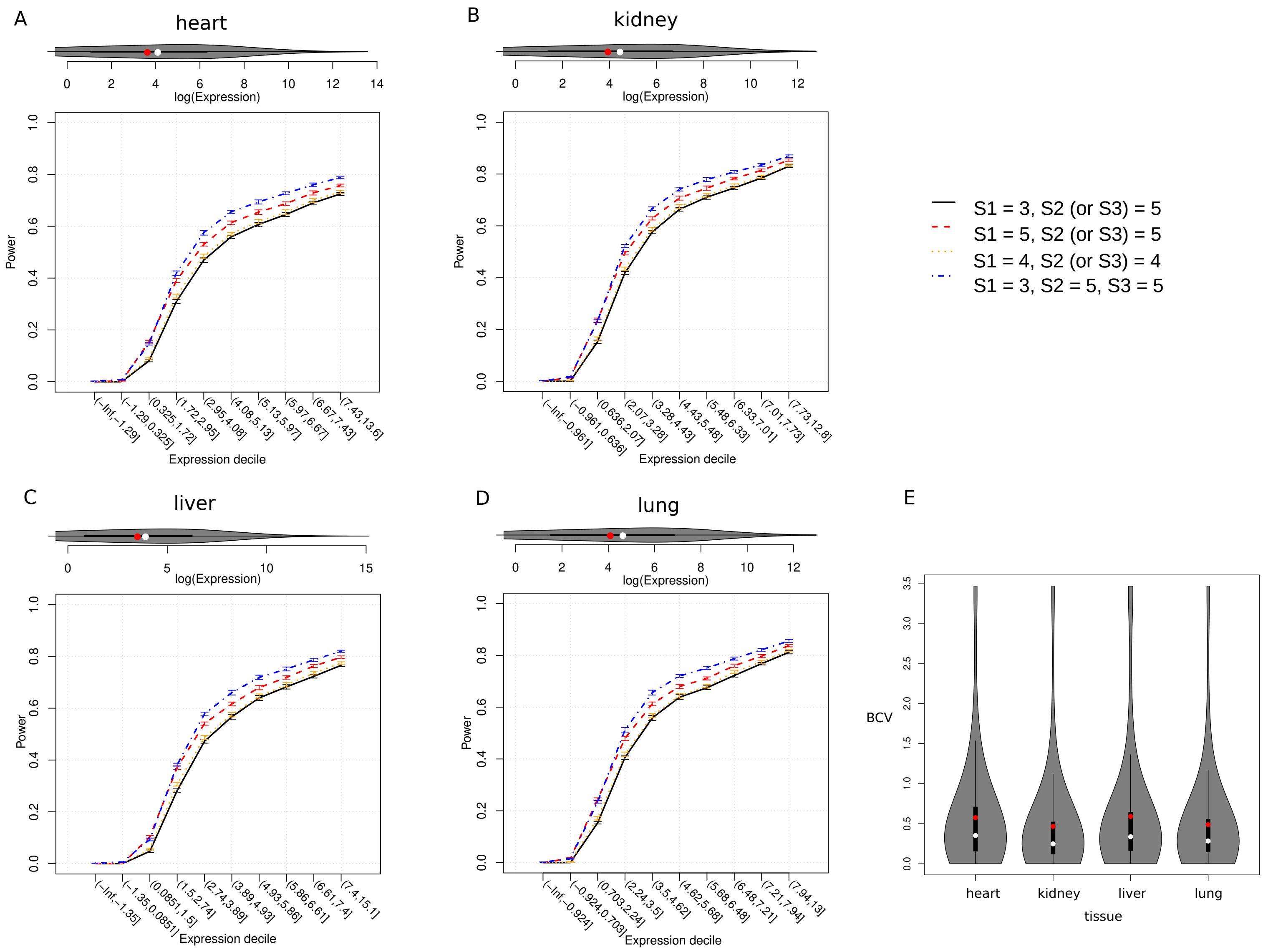
A
heart
log(Expression)
Power
Expression decile
(−Inf,−1.29]
(−1.29,0.325]
(0.325,1.72]
(1.72,2.95]
(2.95,4.08]
(4.08,5.13]
(5.13,5.97]
(5.97,6.67]
(6.67,7.43]
(7.43,13.6]
B
kidney
log(Expression)
Power
Expression decile
(−Inf,−0.961]
(−0.961,0.636]
(0.636,2.07]
(2.07,3.28]
(3.28,4.43]
(4.43,5.48]
(5.48,6.33]
(6.33,7.01]
(7.01,7.73]
(7.73,12.8]
S1 = 3, S2 (or S3) = 5
S1 = 5, S2 (or S3) = 5
S1 = 4, S2 (or S3) = 4
S1 = 3, S2 = 5, S3 = 5
C
liver
log(Expression)
Power
Expression decile
(−Inf,−1.35]
(−1.35,0.0851]
(0.0851,1.5]
(1.5,2.74]
(2.74,3.89]
(3.89,4.93]
(4.93,5.86]
(5.86,6.61]
(6.61,7.4]
(7.4,15.1]
D
lung
log(Expression)
Power
Expression decile
(−Inf,−0.924]
(−0.924,0.703]
(0.703,2.24]
(2.24,3.5]
(3.5,4.62]
(4.62,5.68]
(5.68,6.48]
(6.48,7.21]
(7.21,7.94]
(7.94,13]
E
BCV
heart
kidney
liver
lung
tissue

Supplemental Figure S3: Power of the differential gene expression analysis presented in the primate study, separately for each tissue. A – D: Power values are stratified by deciles of the baseline expression levels. Black solid lines represent a scenario comparing 3 $F_1$ hybrid samples (S1) and 5 pure species samples (S2 or S3). Red dashed lines represent a scenario comparing two species based on 5 samples each (S1 and S2 or S3). Blue dash-dotted lines represent a scenario comparing 3 $F_1$ hybrid samples (S1) and two parental species based on 5 samples each (S2 and S3). Yellow dotted lines represent a scenario comparing two species based on 4 samples each (S1 and S2 or S3, as in REF). Violin plots directly above each panel represent the distribution of expression levels for each tissue, with median values in white and mean values in red. E: Violin plots represent the distribution of the biological coefficient of variation (BCV) separately for each tissue, with median values in white and mean values in red.

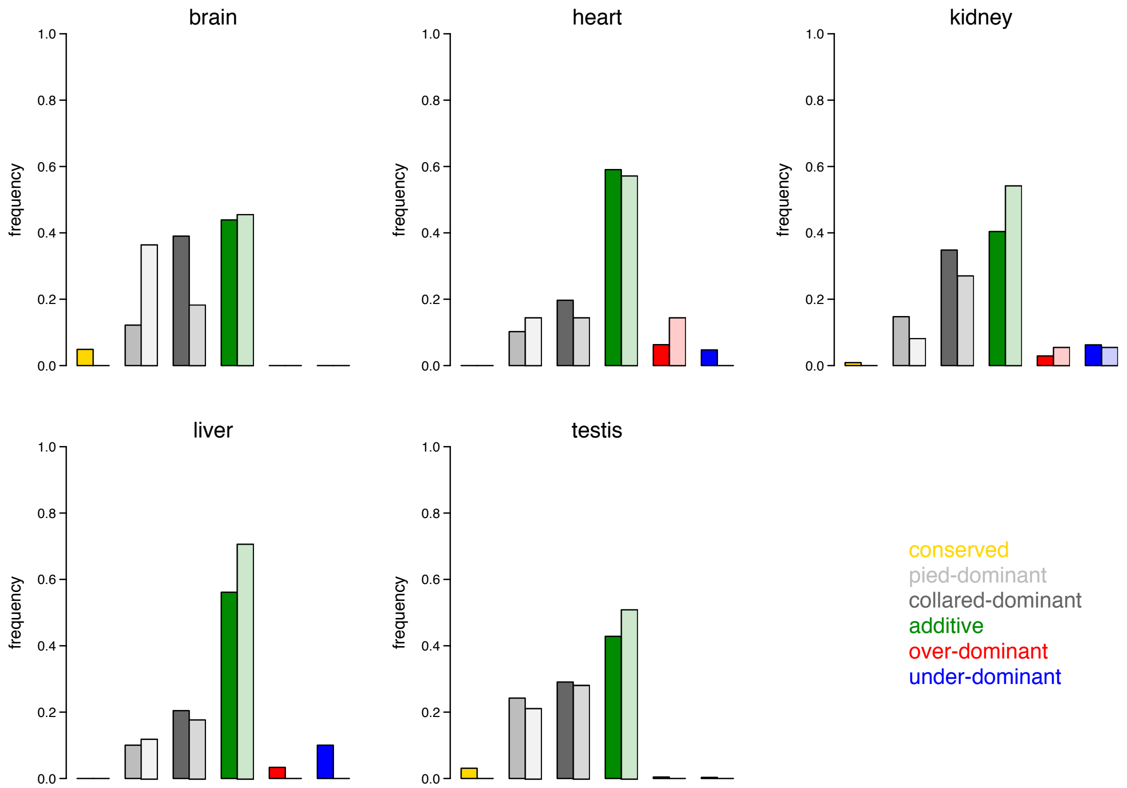


Supplemental Figure S4: Inheritance mode of gene expression patterns for differentially expressed genes across the five tissues, separately for genes located on autosomes (opaque colors) and the Z chromosome (transparent colors). The height of the bars shows the frequency of genes in each of the categories.

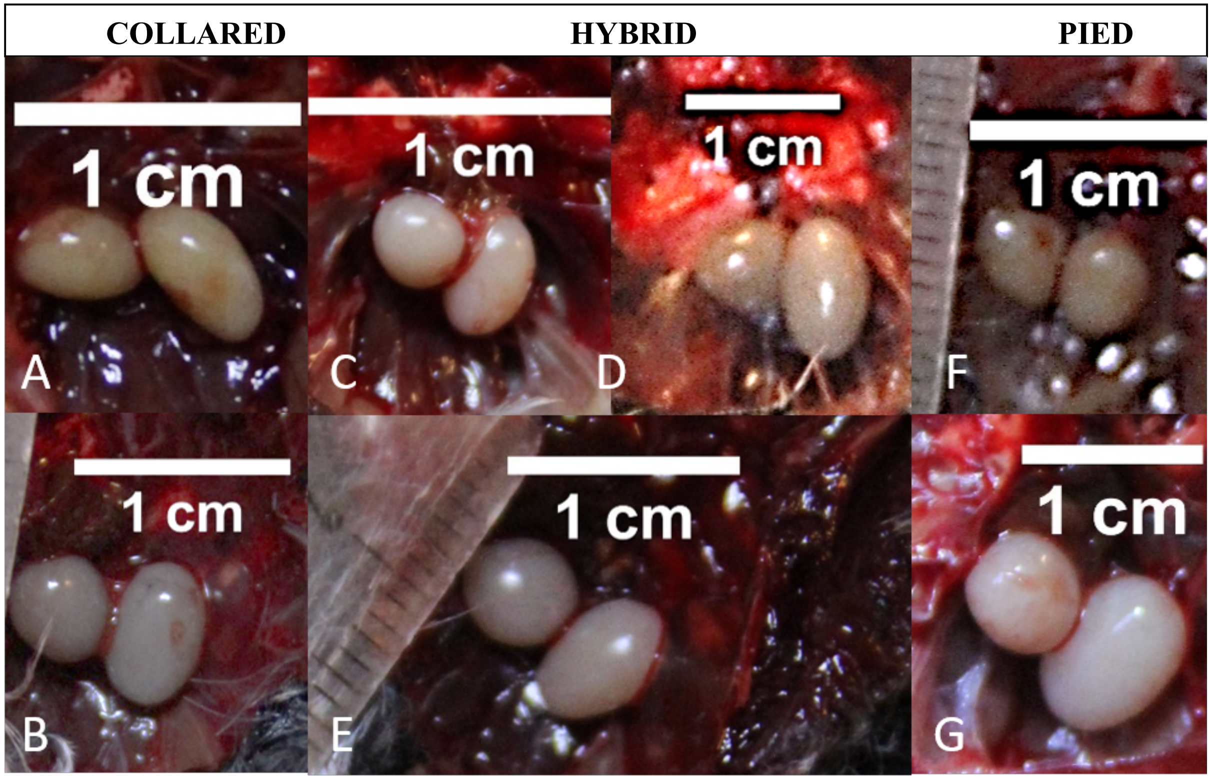


Supplemental Figure S5: Pictures of testes during dissection, while still attached in the body cavity. The testis appearing on the right of each picture is the bird's left testis. A, B: collared flycatchers, C, D, E: $F_1$ hybrid individuals, F, G: pied flycatchers.
